# Discovery of co-stimulatory anti-CD28 VHHs for developing cancer immune therapeutic anti-tumor/CD3/CD28 trispecific T cell engager

**DOI:** 10.3389/fimmu.2026.1812063

**Published:** 2026-04-17

**Authors:** Hui Wang, Rui Zhao, Mengmeng Liu, Tianzhi Jiang, Di Sun, Lida Chi, Yonglin Huang, Zhenghui Lu, Yueli Yun, Xiangbin Wang

**Affiliations:** 1 State Key Laboratory of Biocatalysis and Enzyme Engineering, School of Life Sciences, Hubei University, Wuhan, China; 2 Beijing Scipromed Biotech Co., Ltd., Beijing, China

**Keywords:** agonist, CD28, co-stimulatory, T cell engager, VHH

## Abstract

**Introduction:**

CD28 is a critical co-stimulatory receptor expressed on T cells. Alongside the T-cell receptor (TCR), it provides a dual-signal requirement that is necessary for the complete activation of T cells. Developing co-stimulatory agonists targeting CD28 therefore represents a promising strategy to enhance the efficacy of T cell based anti-tumor immunotherapy.

**Results:**

In this study, we isolated 34 VHHs (VHHs) from a phage-display immune library generated using PBMCs of a Bactrian camel immunized with recombinant human CD28 protein. These VHHs bound to cell surface CD28 with EC_50_ values ranging from 8.5 nM to 130 nM and exhibited varied co-stimulatory activity. When incorporated as building blocks into a trispecific T cell engager (Tri-TCE) targeting DLL3, CD3, and CD28, the anti- CD28 VHHs conferred superior tumor specific cytotoxicity compared to a bispecific T cell engagers (Bi-TCEs) targeting only DLL3 and CD3. Among all candidates, the Tri-TCE containing VHH38 demonstrated the most potent antitumor efficacy. Structural mapping and alanine-scanning mutagenesis revealed that VHH38 engages the CD28 epitope, involving residues Arg34, Tyr51, and Tyr61, a region similar to that recognized by the published superagonistic antibody TGN1412. Unlike TGN1412, however, VHH38 exhibits a lateral paratope orientation that likely spatially hinder the cross-linking of CD28 molecules on the same T cell, probiding a structural basis for its co-stimulatory activity rather than superagonistic activity.

**Discussion:**

In summary, through an integrated process of affinity screening, functional agonism ranking, and therapeutic efficacy evaluation, we have identified a panel of co-stimulatory anti-CD28 VHHs with strong potential as functional modules in Tri-TCEs for cancer immunotherapy.

## 1 Introduction

CD28 is a key co-stimulatory receptor expressed on the surface of T cells (1). It belongs to the type I membrane protein group, and its extracellular region consists of a single domain with a typical immunoglobulin (Ig) fold structure. CD28 has two natural ligands (CD80/CD86) recognizing the unique epitope with the MYPPPY motif (2). Engagement of CD28 by CD80/CD86 delivers a costimulatory signal (Signal 2) that synergizes with the primary antigen-specific signal (Signal 1) provided through the T-cell receptor (TCR)/CD3 complex. This coordinated dual-signal interaction is essential for achieving full T-cell activation, driving robust clonal expansion, cytokine production, enhanced survival, and the development of effector functions (3, 4). However, developing safe and effective CD28 agonists to enhance T cell immunotherapy remains a frontier challenge for researchers. Currently, CD28 agonists can be divided into superagonists and co-stimulatory agonists (5). Superagonists, exemplified by the prototype antibody TGN1412, can potently activate resting T cells independently of Signal 1. This activity is both epitope- and format-dependent, requiring binding to the C’’D loop of CD28 and efficient receptor crosslinking by bivalent IgG molecules, which triggers potent intracellular signaling pathways such as nuclear factor kappa-light-chain-enhancer of activated B cells (NF-κB) (6). The severe cytokine release syndrome and multiorgan failure experienced by all healthy volunteers in the 2006 Phase I trial of TGN1412 starkly highlighted the severe safety risks associated with systemic CD28 superagonism (7). In contrast, conventional agonistic antibodies bind to an epitope nearer the native ligand-binding site. They do not induce autonomous T cell activation but function strictly as co-stimulatory agents, providing a potent co-stimulatory signal only in the context of concurrent TCR engagement (5). Consequently, therapeutic development has pivoted toward engineered CD28 agonists that selectively mimic the physiological, receptor-like signaling of CD80/CD86, aiming to provide safe and controlled co-stimulation (8–10).

Bi-TCEs are engineered immunotherapeutic molecules that redirect T cell cytotoxicity against tumor cells by simultaneously and specifically binding a tumor-associated antigens (TAAs) on tumor cells and the TCR complex on T cell (11–13). To date, ten Bi-TCEs have been approved globally, primarily for hematologic malignancies and, more recently, select solid tumors (14–21). Despite these advances, the efficacy of Bi-TCEs in solid tumors remains limited by poor T cell infiltration, T cell dysfunction, and an immunosuppressive tumor microenvironment (12, 22). Recent research indicates that incorporating T cell co-stimulatory signals into Bi-TCEs to develop novel Tri-TCEs can provide dual activation signals for T cells, significantly enhancing T cell-mediated anti-tumor responses (23, 24). The development of Tri-TCEs employing conventional antibody formats face distinct engineering challenges, including the heavy-light chain mispairing and the instability of single chain variable fragments (scFvs) (25, 26). VHHs, represent the antigen recognizing component of heavy chain only antibodies (HcAbs) naturally occurring in camelids. Unlike conventional antibodies, HcAbs lack light chains, and their antigen-binding capability is mediated solely by a single variable domain (VHH). This single domain architecture confers distinctive advantages, including a small molecular size (approximately 15 kDa), high solubility, exceptional stability under denaturing conditions, and autonomous antigen binding specificity (27–29). These properties make VHHs ideal modular building blocks for engineering artificial multispecific antibodies. To date, three nanobody based therapeutics have received regulatory approval worldwide, demonstrating their translational potential in clinical medicine (30–32). Therefore, developing co-stimulatory VHH agonists targeting CD28 for constructing functional units of Tri-TCEs is a promising endeavor.

This study outlines an integrated discovery strategy for identifying and characterizing co-stimulatory agonistic anti-CD28 VHHs as building blocks for Tri-TCEs. The comprehensive workflow involved five sequential phases. First, a phage-displayed immune VHH library was screened against recombinant human CD28 to isolate initial binders. Second, affinity ranking and cell surface binding validation were performed, selected hits were ranked by their binding affinity to native CD28 expressed on cells using fluorescence activated cell sorting (FACS). Third, the agonistic activity of lead candidates was functionally profiled in a Jurkat cell reporter assay. This step confirmed that the VHHs act as co-stimulatory agonists, activating CD28 signaling primarily in the presence of TCR engagement, thereby distinguishing them from superagonists. Fourth, applicability in a Tri-TCE format was assessed by incorporating representative anti-CD28 VHHs into an anti-DLL3/CD3/CD28 Tri-TCE architecture. The resulting molecules were rigorously tested to confirm target dependent, tumor specific cytotoxicity against DLL3 expressing cancer cells. Finally, epitope mapping was conducted through alanine scanning mutagenesis and complex structure prediction with AlphaFold3. This analysis aims to correlate the observed agonistic functionality with specific structural interactions on the CD28 molecule.

## 2 Materials and methods

### 2.1 Cell lines and culture

All cell lines were maintained at 37 °C in a humidified atmosphere containing 5% CO_2_ under specific culture conditions as follows: FreeStyle™ 293F cells (Thermo Fisher Scientific) were cultured in suspension using serum free KOP293 Ex medium (KAIRUI Biotech) with constant agitation at 120 rpm in a shaker incubator. SHP-77 (ATCC CRL-2195), Jurkat (ATCC TIB-152), and ​CD28-KO-Jurkat (Cyagen) and CD3-KO-Jurkat (Cyagen) cells were grown in RPMI-1640 medium (Hyclone) supplemented with 10% heat inactivated fetal bovine serum (FBS). The Jurkat-Luc reporter cell line (Cobioer Biosciences), engineered to stably express luciferase under the control of IL-2 promoter, was maintained under the same conditions as the parental Jurkat cell line. For functional assays, human peripheral blood mononuclear cells (PBMCs, purchased from miles bio) were cultured short term in RPMI1640 medium supplemented with 10% FBS.

### 2.2 Plasmid vectors

The phagemid vector pSCD2 (33), derived from pHEN2 (MRC Centre for Protein Engineering, Cambridge, UK), and the mammalian expression vector pcDNA3.1 (Thermo Fisher Scientific) served as genetic backbones for constructing VHH expressing constructs and Tri-TCEs in this study.

### 2.3 Immunization and VHH library construction​

A recombinant human CD28 protein fused with a C-terminal polyhistidine tag (CD28-His, ACRO Biosystems) was utilized as the immunogen to immunize a Bactrian camel. The camel received subcutaneous injections of 200 μg immunogen emulsified in complete Freund’s adjuvant (Merck) for the primary immunization, followed by booster injections administered biweekly with the same amount of antigen formulated in incomplete Freund’s adjuvant (Merck). Serum samples were periodically collected, and antibody titers were monitored by indirect ELISA using plates coated with the CD28-His immunogen. Once the serum titer exceeded 1:500,000, PBMCs were isolated from 200 mL of whole blood via density gradient centrifugation using Ficoll reagent (tbdscience, China).

Total RNA was extracted from the PBMCs and reverse transcribed into cDNA. The VHH genes were subsequently amplified by a two-step PCR approach, with primers designed to introduce flanking BspQI restriction sites. The amplified VHH fragments were digested with BspQI (Takara) and cloned into a phagemid vector, pSCD2 (SciProtech, China), which had been engineered from pHEN2 to contain compatible BspQI cloning sites. The resulting library was transformed into *E. coli* TG1 cells (SciProtech, China) for phage display and further bio-panning.

### 2.4 Affinity panning using biotinylated CD28-His

The recombinant CD28-His protein was first biotinylated using an amine reactive biotinylation reagent (e.g., NHS-SS-Biotin, Thermo Fisher Scientific) to enable indirect immobilization on solid supports via Streptavidin Coated 96-Well Plates (beaverbio) for affinity-based selection. Three iterative rounds of affinity panning were performed under progressively stringent conditions to enrich for high affinity binders. The amount of CD28-His used per round was systematically reduced: 1 µg in the first round, 0.5 µg in the second round, and 0.1 µg in the third round. This reduction in antigen concentration serves as a critical selection pressure, favoring the enrichment of phage clones with higher binding affinity and specificity by limiting antigen availability and increasing washing stringency between rounds.

Following the third round of biopanning, clones were randomly selected from the elution plate. Each clone was subjected to phage amplification in a 96 well deep plate format to produce phage particles. The supernatants containing the amplified phage particles were then screened using a phage enzyme linked immunosorbent assay (phage ELISA) to identify clones exhibiting specific binding to the target antigen. Clones demonstrating significant positive signals in the phage ELISA were considered putative binders. The plasmid DNA of these positive clones was extracted and subjected to DNA sequencing to determine the nucleotide sequences of the inserted VHH genes. The resulting amino acid sequences were analyzed to find unique clones for further functional characterization.

### 2.5 Preparation of VHH-Fc fusion proteins​

The VHH genes corresponding to the unique positive clones were inserted into a pcDNA3.1 vector pre-encoding the Fc domain of human IgG1, enabling the expression of bivalent VHH-Fc fusion proteins. Transient transfection was performed using FreeStyle™ 293-F cells as the host system. Following incubation, culture supernatants were harvested and subjected to affinity purification via Protein A chromatography (Biolink). The eluted fractions were subsequently analyzed by both SDS-PAGE and size exclusion-high performance liquid chromatography (SEC-HPLC) to assess purity, oligomeric state, and integrity of the fusion proteins.

### 2.6 Ranking of binding affinity by flow cytometry

The relative binding affinity of each unique positive clone was ranked based on the half-maximal effective concentration (EC_50_) derived from saturation binding curves. The assays were performed using Jurkat cells, which express endogenous CD28 on their surface. Serial dilutions of each VHH-Fc fusion protein were incubated with Jurkat cells to allow for equilibrium binding. Cell-bound VHH-Fc was subsequently detected using an Alexa Fluor^®^ 647-conjugated goat anti-human Fc secondary antibody (Jackson ImmunoResearch). Fluorescence was measured using a BD Accuri™ C6 Plus (BD), and the mean fluorescence intensity (MFI) at each concentration was used to generate binding curves. The EC_50_ values, representing the concentration of VHH-Fc required to achieve 50% of maximal binding, were calculated through nonlinear regression analysis of these curves, providing a quantitative metric for comparative affinity ranking among the selected clones.

### 2.7 Detection of co-stimulatory activity using Jurkat-Luc reporter cells

The co-stimulatory agonistic activity of anti-CD28 VHH-Fc fusion proteins was evaluated using Jurkat-Luc reporter cells engineered to express an inducible luciferase gene. Briefly, 10 μg/mL anti-CD28 antibody and 0.1 μg/mL anti-CD3 mAb (OKT3, SciProtech), were coated in PBS onto 96-well plates overnight at 4 °C. Control wells were coated with anti-CD28 antibody alone, anti-CD3 mAb alone, or PBS only. After washing with PBS, Jurkat-Luc reporter cells were seeded into the plates and incubated at 37 °C with 5% CO_2_ for 6 hours. Subsequently, cell lysates were prepared, and luciferase activity was measured by adding a commercial luciferase substrate (Cobioer Biosciences). Luminescence signals were quantified using a microplate reader, with NFAT activation reflected by relative luminescence units (RLU). The method for further evaluation of candidate molecules was similar, except that the antibody concentration was adjusted to serially diluted anti-CD28 antibody and 1 μg/mL OKT3.

### 2.8 Design, expression, and purification of anti-DLL3/CD3/CD28 Tri-TCE

The anti-DLL3/CD3/CD28 Tri-TCE was designed as a two-chain molecule assembled via a knob-into-hole (KiH) Fc heterodimerization platform to ensure correct chain pairing. The first chain comprised an anti-DLL3 scFv, an anti-CD3 scFv, and a human IgG1 Fc fragment with the “Knob” mutation (T366W). The functional units of these two antibodies are derived from Tarlatamab, a Bi-TCE drug that is already on the market. The second chain contained an anti-CD28 VHH domain fused to a human IgG1 Fc fragment bearing the “Hole” mutations (T366S, L368A, Y407V). To eliminate Fcγ receptor (FcγR) binding, the “LALA” mutation was introduced into the Fc region of both chains.

DNA sequences encoding each chain were individually cloned into a pcDNA3.1 vector. The two plasmids were co-transfected into FreeStyle ™293-F cells at a 1:1 mass ratio using polyethylenimine (PEI). Cells were cultured in serum-free medium for 7 days at 37 °C with 8% CO_2_ and constant agitation.

The Tri-TCE protein was purified from clarified culture supernatant by Protein A affinity chromatography (BioLink), followed by cation-exchange chromatography (CEX) as a polishing step to remove aggregates and contaminants. The final product was analyzed by SDS-PAGE to confirm subunit molecular weight and disulfide bonding, and by SEC-HPLC to assess purity, oligomeric state, and aggregation levels under native conditions.

### 2.9 Cytotoxicity assay

Briefly, the carboxyfluorescein succinimidyl ester (CFSE, Thermo Fisher)-labeled target SHP-77 cells (ATCC) were co-cultured with human PBMCs at an effector-to-target (E:T) ratio of 1:5 in 96-well plates (PBMCs for each experiment were sourced from the same donor). Serial dilutions of the Tri-TCE construct were added to the co-cultures. Control wells containing target cells and PBMCs received complete RPMI medium alone to assess background cell death. After 7 days of incubation at 37 °C in a humidified 5% CO_2_ atmosphere, cells were harvested and stained with 7-aminoactinomycin D (7-AAD, Thermo Fisher). Samples were acquired on a CytoFLEX flow cytometer (Beckman) and data were analyzed using CytExpert software (Beckman). The percentage of specific cytotoxicity was calculated using the following formula:

% Cytotoxicity = (A_sample_ −A_spontaneous_) (A_maximum_ −A_spontaneous_)×100%.

where: A_sample_ is the fluorescence reading (percentage of 7-AAD^+^ cells) in wells containing target cells, effector cells, and the Tri-TCE construct; A_spontaneous_ is the fluorescence in control wells containing target and effector cells without Tri-TCE (spontaneous release); A_maximum_ is the fluorescence in wells where target cells were completely lysed (e.g., with Triton X-100) to represent maximum release. This formula was applied to quantify the Tri-TCE-induced specific cytotoxicity in each experimental condition.

Tumor antigen-dependent cytotoxicity was evaluated using SHP77 and RKO-E6 cell lines. Additionally, a slight modification was applied to the method: an effector-to-target ratio of 5:1 and an incubation time of 48 hours were used.

### 2.10 Alanine scanning mutagenesis for binding epitope mapping

To systematically characterize the functional binding epitope of the anti-CD28 VHHs, alanine scanning mutagenesis was employed. Based on the three-dimensional structure of CD28 predicted by AlphaFold3, solvent-exposed residues were selected for individually mutated to alanine to assess its contribution to nanobody binding while minimizing structural perturbations to the protein fold. The preparation process of each mutant protein is briefly described as follows: a poly-histidine tag was added to the C-terminus of each mutant- human serum albumin (HSA) fusion protein, and the same expression method as VHH-Fc was used, but the purification step was performed by Ni-TED chromatography.

Binding interactions between the alanine mutants and anti-CD28 VHHs were assessed using an enzyme-linked immunosorbent assay (ELISA). The 96-well microplate (Corning) were coated with 1 μg/mL of recombinant CD28 antigen (or its alanine variants) in phosphate buffered saline (PBS) and incubated overnight at 4 °C. Plates were then blocked with 4% (w/v) skim milk powder in PBS containing 0.05% Tween-20 (PBST) for 1 hour at 37 °C to prevent nonspecific binding. After washing three times with PBST, serially diluted VHH-Fc fusion proteins were added to the plates and incubated for 1 hour at 37 °C. Following another washing step, bound VHHs were detected using an HRP-conjugated goat anti-human Fc secondary antibody (Jackson ImmunoResearch) diluted in blocking buffer. After a final wash, tetramethylbenzidine (TMB) substrate (Servicebio) was added, and the reaction was stopped with 1 M sulfuric acid. Absorbance was measured at 450 nm using a microplate reader (BioTek), and binding curves were analyzed to determine EC_50_ values for each mutant compared to the wild type CD28 protein.

### 2.11 Complex structure prediction and visualization

To predict the interaction mode between the anti-CD28 nanobody (VHH) and the CD28 antigen, the structural complex was modeled using AlphaFold3, a deep learning-based tool renowned for its high accuracy in predicting biomolecular complexes (34). The amino acid sequences of the CD28 antigen and the selected anti-CD28 VHH were submitted as inputs to the publicly accessible AlphaFold3. The top-ranked structural model generated by AlphaFold3, which is accompanied by the highest predicted confidence score (pTM or iPTM), was downloaded for subsequent analysis. This model was imported into PyMOL for visualization and analysis.

## 3 Results

### 3.1 Generation of anti-human CD28 VHHs

**Figure 1:**
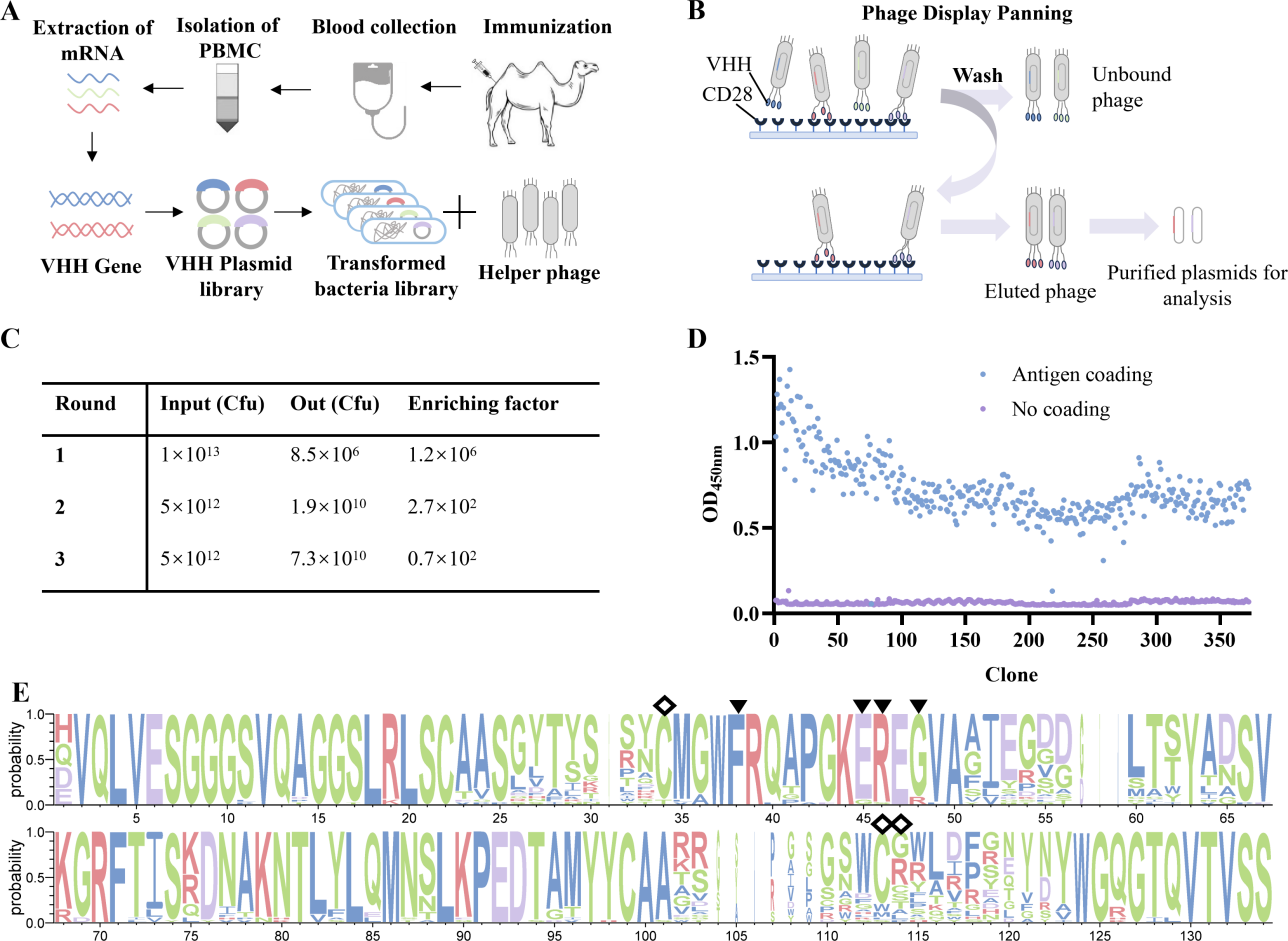
Generation and analysis of anti-CD28 VHHs. **(A)** Phage display library construction process. Bactrian camels were immunized with the CD28 extracellular domain. Peripheral blood mononuclear cells (PBMCs) were isolated, followed by RNA extraction and cDNA synthesis. VHH gene fragments were amplified and cloned to construct a phage‑display library. **(B)** Biological screening process. Specific binders were isolated through solid‑phase biopanning and validated by monoclonal phage ELISA. **(C)** Process parameters for solid-phase panning. **(D)** Results of phage ELISA identification. **(E)** Sequence analysis of anti-CD28 VHH. The conservation of amino acid sites in 34 VHHs was analyzed using Weblogo3 (36). Hallmark regions are marked with triangles (▼), while cysteine sites that form non-classical disulfide bonds are marked with rhombuses (◇).

Upon confirmation that the immune serum from the immunized Bactrian camel exhibited a titer exceeding 1:500,000, a phage-displayed VHH library was constructed, with a capacity of 2.3 × 10^9^ independent transformants and a positive rate of 90% (**Figure 1A**). Three rounds of biopanning were performed on the CD28 immobilized extracellular domain, with enrichment factors of 1.2 × 10^6^, 2.7 × 10^2^, and 0.7 × 10^2^, respectively, significantly enriching antigen-specific phages (**Figures 1B, C**). From the third-round eluate, 372 individual clones were randomly selected for phage ELISA screening, which identified a number of positive clones. Nucleotide sequencing of these positive clones yielded 95 unique VHH amino acid sequences (**Figure 1D**). Sequences with the same complementarity-determining region 3 (CDR3) were excluded, and 34 representative VHHs were chosen for subsequent construction, expression, and purification as VHH-Fc fusion proteins.

All 34 unique VHH sequences featured a characteristically long CDR3 loop, typically comprising 15–21 amino acid residues, which aligns with the canonical structural features of camelid-derived VHHs (**Figure 1E**). Among these, 31 clones contained the conserved “FERG” motif in framework region 2 (FR2), a hallmark known to enhance the hydrophilicity and solvent compatibility of VHHs compared to the VH domains of conventional antibodies (35). Furthermore, 32 clones possessed cysteine residues within their CDR3 regions (**Supplementary Figure 1**). These cysteine residues form intra-domain disulfide bonds with cysteine residues located in CDR1, a structural adaptation that stabilizes the extended CDR3 loop and shields hydrophobic amino acids that would otherwise be exposed due to the absence of a paired light chain.

### 3.2 Affinity-based ranking of 34 positive clones to cell surface CD28

**Figure 2:**
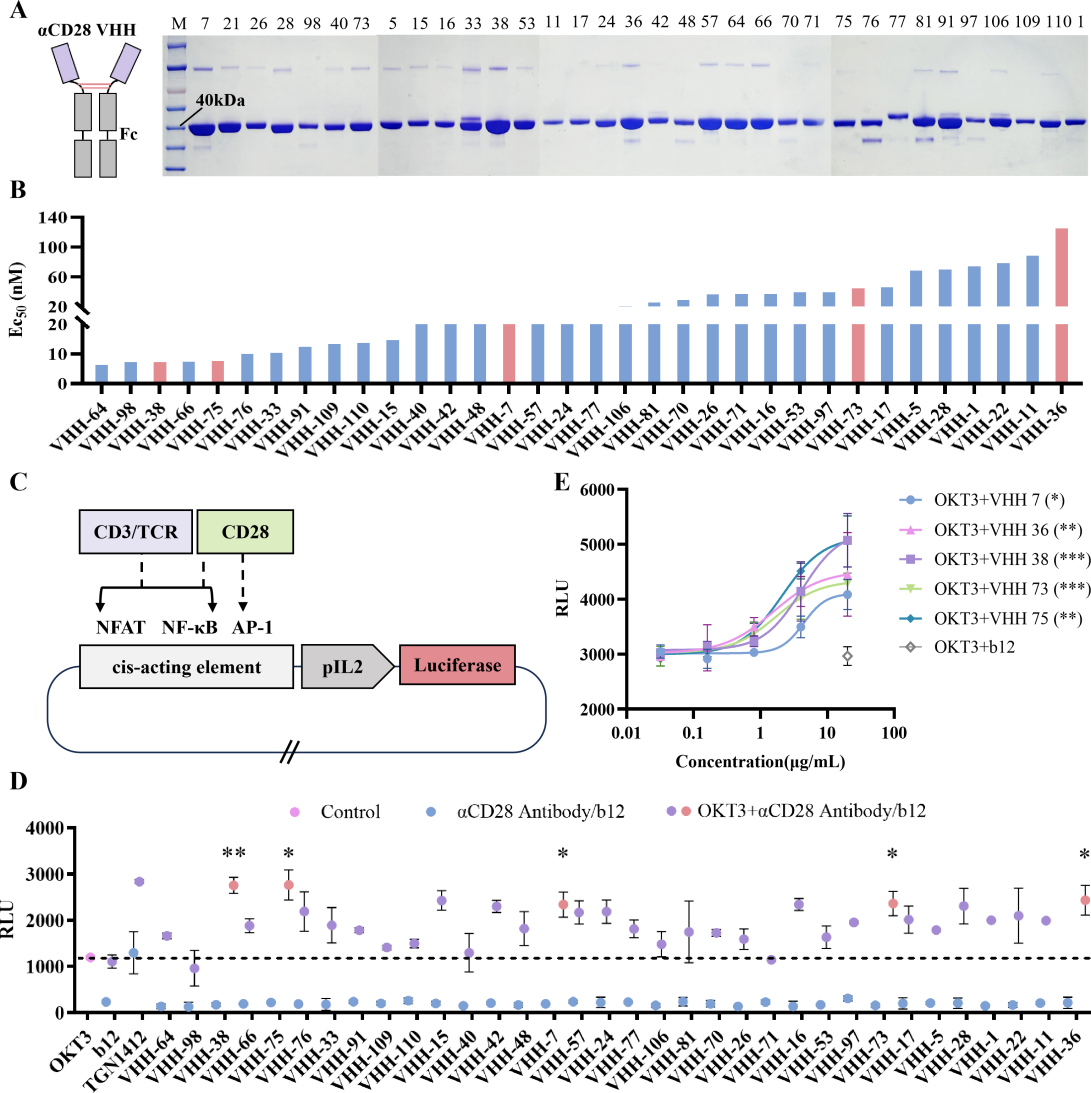
Preparation and characterization of VHH-Fc. **(A)** SDS-PAGE analysis of purified VHH-Fc. The left image shows the molecular form of VHH-Fc. In the right image, the VHH-Fc number is above the SDS-PAGE. The theoretical molecular weight of the VHH-Fc is approximately 39 kDa. **(B)** The binding capability of VHH-Fc to surface-expressed CD28 on Jurkat cells was evaluated using FACS method. Cell surface-bound VHH-Fc were detected by Alexa Fluor^®^ 647-conjugated goat anti-human Fc antibody. Mean fluorescence intensities were determined by flow cytometry assay. **(C)** Luciferase regulatory elements in Jurkat-Luc reporter cells. CD3/TCR and CD28 can regulate IL2 promoter transcription through signaling pathways such as NFAT, NF-κB, and AP-1, thereby initiating Luciferase gene expression. **(D)** Luciferase reporter assay for CD28 VHH agonistic activity. Reporter cells were stimulated with the indicated antibodies either alone or in combination with a fixed concentration of OKT3 (0.1 μg/mL). The resulting agonistic effect was quantified by luciferase activity. TGN1412 and an irrelevant antibody, b12 (anti-HIV-1 gp120), served as controls. VHH antibodies marked in red (VHH7, VHH36, VHH38, VHH73, and VHH75) were identified as strong co-stimulatory agonists. A two-tailed unpaired t-test was used to compare the RLU between the OKT3+CD28 VHH and the OKT3 control (n = 3 independent experiments). ∗*P* < 0.05, ∗∗*P* < 0.01, ∗∗∗*P* < 0.001. **(E)** Assess the agonistic activity of representative anti-CD28VHH by Jurkat-Luc reporter cell. Reporter cell luciferase activity was measured under stimulation with OKT3 (1 μg/mL) in combination with various antibody concentrations (n=3 independent experiments). Data are expressed as mean ± SD. A two-tailed unpaired t-test was used to compare the RLU at the maximum dose between OKT3+CD28 VHH and the OKT3+b12 control group (n = 3 independent experiments). ∗*P* < 0.05, ∗∗*P* < 0.01, ∗∗∗*P* < 0.001.

VHH-Fc fusion proteins derived from the 34 unique positive clones were successfully expressed and purified (**Figure 2A**). The quality of each fusion protein was confirmed by SDS-PAGE and SEC-HPLC to ensure integrity and mono-dispersity prior to functional evaluation. The results showed that each molecule had a purity of over 90%, indicating that all molecules were producible. The binding affinity of each clone to native, membrane-anchored CD28 was assessed using Jurkat cells, which express endogenous CD28 on their surface. All 34 VHH-Fc constructs demonstrated specific binding to Jurkat cells, with EC_50_ values spanning a range from 7 nM to 130 nM, indicating a nearly 17-fold difference between the highest and lowest affinity binders (**Figure 2B**). This quantitative ranking provided a clear affinity gradient for downstream selection.

### 3.3 Identification of five co-stimulatory anti-CD28 VHHs via a Jurkat-Luc reporter cell assay

To functionally characterize the selected anti-CD28 VHHs, we employed a Jurkat-Luc reporter cell assay. In this system, luciferase expression is driven by a promoter containing nuclear factors of activated T cells (NFAT), NF-κB and activator protein 1 (AP-1) response elements, enabling quantitative measurement of T cell activation through luminescence output (**Figure 2C**). A critical control was implemented to discriminate co-stimulatory from superagonists: the assay was also performed in the absence of OKT3. Initial screening results showed that TGN1412 alone could induce luminescence, and when used concurrently with OKT3, it enhanced the luminescence induced by the latter. In contrast, all anti-CD28 VHHs only enhanced luminescence when used in combination with OKT3, and did not induce luminescence when used alone. Based on this result, we preliminarily conclude that all anti-CD28 VHHs possess co-stimulatory properties (**Figure 2D**). To select representative VHHs for further characterization, a rational multi-parameter filtering process was applied rather than random selection. First, candidates were required to rank in the top 50% for expression yield to ensure robust manufacturability (**Supplementary Table 1**). Second, they had to exhibit less than 80% sequence similarity in the CDR3 region to maximize diversity (**Supplementary Figure 2**). Third, we intentionally selected candidates with diverse affinity and strong agonistic activity, because agonistic activity is not always linearly correlated with affinity (**Figures 2B, D**). Finally, to minimize structural and developability liabilities, all selected candidates possessed an alkaline isoelectric point (pI) for favorable stability and were completely devoid of potential N-linked glycosylation sites (N-X-S/T motifs) (**Supplementary Figure 1**). Based on the above principles, five representative clones, VHH7-Fc, VHH36-Fc, VHH38-Fc, VHH73-Fc, and VHH75-Fc, were selected for further functional characterization. This selection ensured coverage of a broad affinity spectrum and CDR3 sequence divergence for further screening of subsequent agonistic activities.

Further results showed that the five VHH-Fc fusion proteins significantly enhanced luminescence only when used in combination with OKT3, with VHH75 and VHH38 exhibiting stronger agonistic activity (**Figure 2E**). Conversely, none induced detectable T cell activation when applied alone. This strict dependence on TCR co-stimulation definitively classifies all five candidates as co-stimulatory anti-CD28 VHHs, not superagonists. This functional stratification is essential for downstream therapeutic development, as co-stimulatory agonists, which require a signal 1, typically offer a superior safety profile by mitigating the risk of uncontrolled cytokine release associated with superagonistic activity.

### 3.4 Evaluation of anti-CD28 VHHs in Tri-TCEs

**Figure 3:**
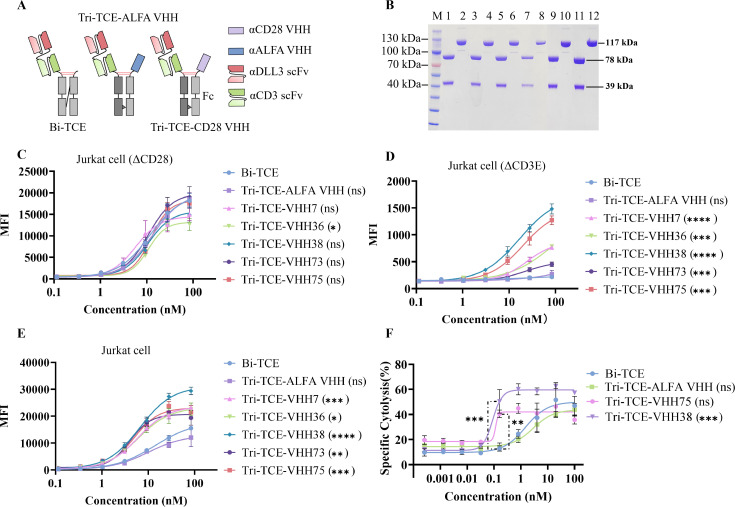
Design, expression, purification and functional characterization of Tri-TCEs. **(A)** The format of Tri-TCEs and Bi-TCE. **(B)** SDS-PAGE analysis of purified Tri-TCEs. Lanes 1, 3, 5, 7, 9, and 11 represent the purified Tri-TCE-VHH7, Tri-TCE-VHH36, Tri-TCE-VHH38, Tri-TCE-VHH73, Tri-TCE-VHH75, and Tri-TCE-ALFA VHH detected under reducing (R) conditions, respectively. Lanes 2, 4, 6, 8, and 10 represent the results detected under non-reducing (NR) conditions. The theoretical molecular weights of the two chains and the complete antibody molecule are 39 kDa, 78 kDa, and 117 kDa, respectively. **(C)** FACS analysis of the binding ability of TCE to CD3ε on CD28KO Jurakt cells. Cell surface-bound TCE were detected by Alexa Fluor® 647-conjugated goat anti-human Fc antibody. Mean fluorescence intensities were determined by flow cytometry assay. **(D)** FACS analysis of the binding ability of TCE to CD28 on CD3ε KO Jurakt cells. Cells were stained with the same protocol as (C). **(E)** FACS analysis of the binding ability of TCE to both CD3ε and CD28 on Jurakt cells. Cells were stained with the same protocol as (C). A two-tailed unpaired t-test was used to compare the MFI at the maximum dose between the Tri-TCE and the Bi-TCE control (n = 3 independent experiments). ∗P < 0.05, ∗∗P < 0.01, ∗∗∗P < 0.001. **(F)** TCE mediated tumor cell killing by PBMCs. Cytotoxic activity of PBMCs against SHP77 cells in the presence of the indicated antibodies. The tumor target cells cocultured with human PBMCs at an effector-to target ratio of 1:5. Effector cytolytic activity was assessed after 7 days. The data shown here comes from the same donor. All data shown here are the mean ±SD values from three independent triplicates values. A two-tailed unpaired t-test was used to compare the cytotoxicity at the maximum dose (or 0.16nM) between the Tri-TCE and the Bi-TCE control (n = 3 independent experiments). ∗P < 0.05, ∗∗P < 0.01, ∗∗∗P < 0.001.

Tri-TCEs represent an advanced class of artificial antibodies engineered to recruit and activate T cells by simultaneously engaging multiple antigens. However, the incorporation of three antigen-binding domains can introduce steric constraints that may impair functionality. To assess the applicability of the selected anti-CD28 VHHs within a Tri-TCE format, we constructed five CD3/CD28/DLL3-targeting Tri-TCE molecules (**Figure 3A**). These were evaluated alongside two control constructs: a CD3/ALFA/DLL3 Tri-TCE (Tri-TCE-ALFA VHH), in which the CD28 VHH was replaced by an ALFA-specific VHH targeting an inert peptide (37), and a CD3/DLL3 Bi-TCE based on the clinically approved drug tarlatamab for small-cell lung cancer (38).

All constructs were expressed in FreeStyle ™293-F cells via transient transfection and purified to >95% homogeneity, as confirmed by SEC-HPLC and SDS-PAGE (**Figure 3B**). Given that the anti-DLL3 domain was positioned at the N-terminus to minimize steric interference, we first evaluated the binding of the anti-CD3 and anti-CD28 domains within the Tri-TCEs to their cognate cell-surface antigens. Using CD28 knockout Jurkat cells, we demonstrated that all Tri-TCEs bound CD3 with EC_50_ values ranging from 5 to 15 nM, which was comparable to both the Bi-TCE control (11.5 nM) and the Tri-TCE-ALFA VHH (13.1 nM), indicating no significant impairment in CD3 engagement due to the trispecific format (**Figure 3C**). Assessment of CD28 binding using CD3 knockout Jurkat cells revealed variations relative to the bivalent VHH-Fc format. Among the trispecific molecules, Tri-TCE-VHH38 exhibited the strongest binding (EC_50_ = 16.8 nM), while Tri-TCE-VHH73 (EC_50_ = 23.4 nM) and Tri-TCE-VHH7 (EC_50_ = 24.1 nM) showed moderate reductions in affinity or maximal binding signal (**Figure 3D**). These differences likely reflect variations in epitope accessibility and structural constraints imposed by the trispecific architecture. Notably, all CD28 containing Tri-TCEs exhibited higher maximum binding to wild type Jurkat cells compared to either the Bi-TCE or the Tri-TCE-ALFA VHH control (**Figure 3E**), demonstrating that the incorporated anti-CD28 VHHs functionally enhance T cell binding without compromising structural integrity.

**Figure 4:**
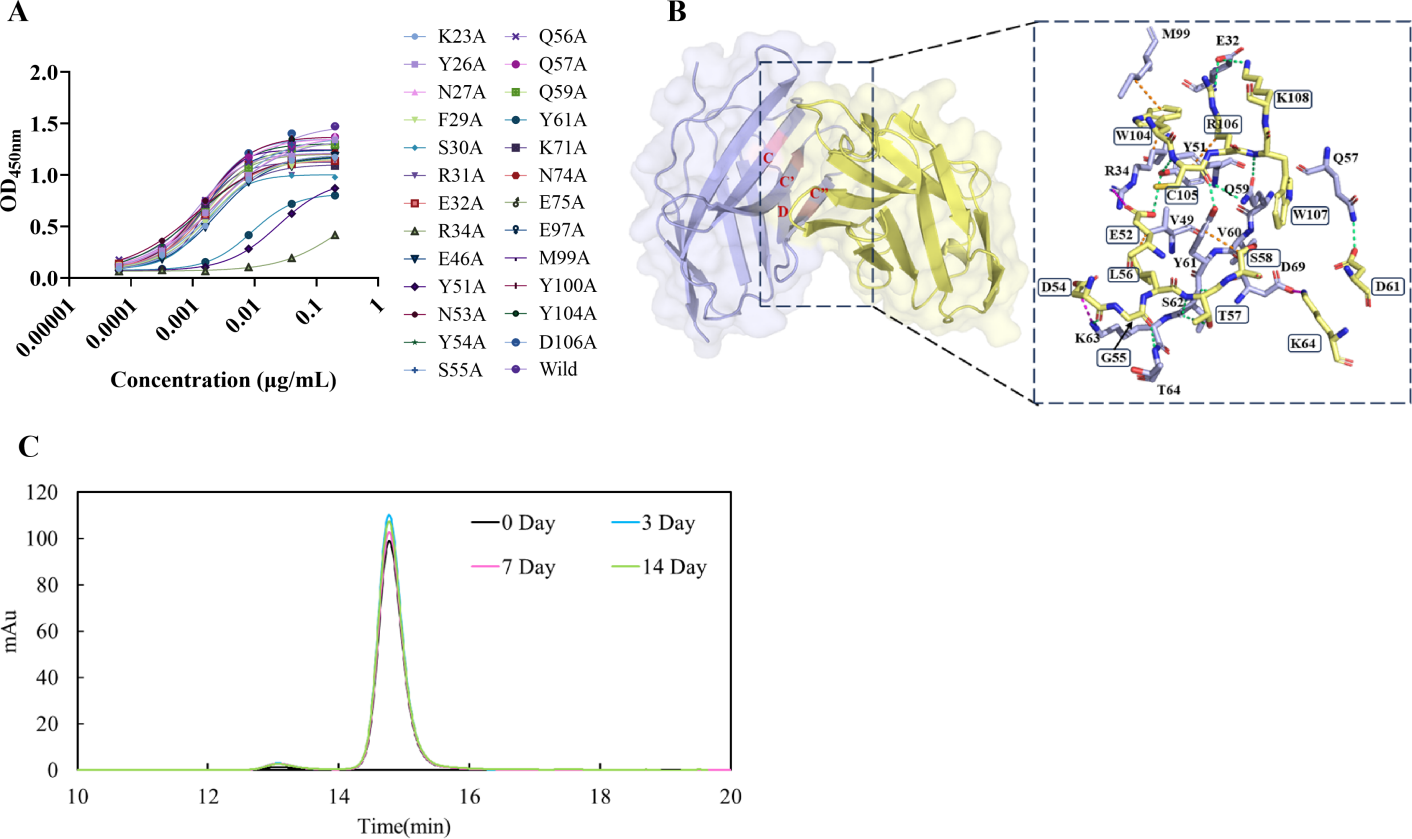
Epitope mapping of VHH38 and the thermal stability of Tri-TCE-VHH38. **(A)** Detection of the binding ability between VHH38 and CD28 mutants. An ELISA was performed to assess the binding of VHH38-Fc to a panel of CD28 mutants. Binding was detected with an HRP-conjugated goat anti-human Fc secondary antibody. The results indicate that alanine substitutions at R34, Y51, and Y61 markedly decreased the binding. **(B)** Analysis of the interaction interface between CD28 and VHH 38. VHH38 and CD28 are marked in yellow and purple, respectively. VHH38 residues are highlighted in black boxes; hydrogen bonds, salt bridges, and hydrophobic contacts are indicated in green, magenta, and orange, respectively. **(C)** Thermal stability assessment of Tri-TCE-VHH38. Evaluation using SEC-HPLC showed that the sample quality did not decrease significantly after incubation at 37°C for 14 days.

Based on their favorable binding characteristics, Tri-TCE-VHH38 and Tri-TCE-VHH75 were selected for cytotoxicity evaluation against tumor cells. As shown in **Figure 4**, both the Bi-TCE and the Tri-TCE-ALFA VHH control induced similar levels of target cell lysis, with comparable EC_50_ values around 1.49 nM. In contrast, Tri-TCE-VHH75 and Tri-TCE-VHH38 significantly enhanced PBMC dependent cytotoxicity in a dose-dependent manner, with EC_50_ values of 0.12 nM and 0.09 nM, respectively (**Figure 3F**). The steep dose-response curve between 0.05 and 0.1 nM, followed by a rapid plateau, reflects the characteristic threshold of immunological synapse formation and subsequent receptor saturation typical of highly potent T cell engagers. Notably, Tri-TCE-VHH38 demonstrated the most potent tumor-killing activity, showing a 16.6-fold reduction in EC_50_ and a 1.4 fold increase in maximal cytotoxicity compared to the Bi-TCE control. Together, these findings confirm that Tri-TCE-VHH38 outperforms the Bi-TCE across multiple functional parameters, including T cell binding and tumor cell cytotoxicity.

### 3.5 Determination of the VHH38 binding epitope region and structural characterization of the complex

Given the superior functional performance of Tri-TCE-VHH38, we sought to characterize its binding epitope on CD28 through systematic alanine scanning mutagenesis. A panel of CD28 mutants was generated by individually substituting solvent exposed residues in the extracellular domain with alanine. Each mutant was fused to HSA to enhance stability and expressed in FreeStyle ™293-F. Binding affinity between the mutant CD28-HSA fusion proteins and VHH38-Fc was quantified using ELISA (**Figure 4A**). Mutations R34A, Y51A, and Y61A dramatically reduced binding affinity, with R34A most severely impairing binding, as reflected by a 100-fold increase in EC_50_ relative to wild type CD28.

To elucidate the structural basis of this interaction, we employed Alphafold3, a deep learning-based framework that integrates diffusion and AlphaFold2 modules for high-accuracy protein complex structure prediction. Models with both TM score and pTM score exceeding 0.6 were selected as high-confidence predictions (34). The resulting VHH38/CD28 complex structure revealed an interface stabilized by 12 hydrogen bonds, alongside significant electrostatic and hydrophobic contributions (**Figure 4B**). Key interactions include eight hydrogen bonds formed by CDR2 residues Glu52, Asp54, Gly56, Thr57, Ser58 and Asp61 interacting with Tyr51, Lys63, Thr64, Ser62, Val60 and Gln57 of CD28, respectively. Four additional hydrogen bonds involve CDR3 residues Cys105 and Typ109 of VHH38 with Tyr51, Gln59 and Tyr61 of CD28, respectively. Beyond hydrogen bonding, the interface is further stabilized by electrostatic and hydrophobic interactions. Glu52 (CDR2), Asp54 (CDR2), Lys64 (CDR2), Arg106 (CDR3) and Lys108 (CDR3) of VHH38 form five electrostatic pairs with Arg34, Lys63, Asp69 and Glu32 of CD28, respectively. Residues Ser58 (CDR2), Trp104 (CDR3) and Arg106 (CDR3) engage in alkyl-π interactions with Tyr61, Arg34, Met99 and Tyr51 of CD28, while Leu56 (CDR2) forms a hydrophobic contact with Val49 of CD28. Notably, Arg34​ residue ​of CD28 participated in multiple interaction types: electrostatic pairing with Glu52 and alkyl-π stacking with Trp104. These combined interactions likely underlie the severe binding deficit caused by the R34A mutation. These results collectively indicate that the VHH38 epitope is primarily localized to the C and C’ *β*-sheets and the C’’ D loop of CD28, providing a structural rationale for its functional efficacy.

## 4 Discussion

The clinical application of CD28 agonists has been marred by significant risks, as exemplified by the severe cytokine release syndrome induced by the superagonistic anti-CD28 antibody TGN1412. Unlike co-stimulatory agonists, superagonists can activate T cells independently of TCR ligation, leading to uncontrolled polyclonal T cell expansion and a dangerous systemic inflammatory response. This underscores a critical unmet need for co-stimulation CD28 agonistic antibodies​ whose activation capacity is strictly contingent upon concurrent TCR engagement, thereby mirroring the physiological activation pathway and providing better safety for T-cell-based immunotherapies (1, 8, 10, 39).

Bi-TCEs has encountered challenges in treating solid tumors, mainly because solid tumors have a highly immunosuppressive tumor microenvironment (TME), which not only restricts T cell infiltration but also weakens T cell function through multiple mechanisms (40). On the one hand, the downregulation of CD80/CD86 expression on tumor cells and dendritic cells (DCs) leads to the loss of co-stimulatory signals necessary for T cell activation. On the other hand, the upregulation of immune checkpoint molecules on T cells further induces T-cell exhaustion or incapacitation (41). Developing Tri-TCE for the treatment of solid tumors, based on the classic T-cell dual-signal activation theory, is a promising direction. Tri-TCEs introduces an agonist targeting CD28, building upon the traditional CD3×TAA dual-targeting Bi-TCE. This design allows Tri-TCEs to simultaneously provide Signal 1 and Signal 2 when bridging tumor cells and T cells. By providing this dual-signal activation, Tri-TCEs not only potentiate T cell activation and cytotoxicity but also help delay T cell exhaustion and improve response durability, offering a promising strategy to conquer the immunosuppressive barriers in solid tumors (42, 43).

To address this need, we isolated a panel of 34 anti-CD28 variable VHHs from a phage display library derived from an immunized camelid. Functional assays confirmed the desired therapeutic profile of these selected VHHs. They potently enhanced downstream signaling and luciferase reporter activity initiated by the CD3/TCR complex when co-stimulated with a suboptimal concentration of an anti-CD3 antibody. Most importantly, and in stark contrast to superagonists, these VHHs exhibited no capacity to activate T cells when administered alone. This demonstrates their strict dependence on TCR co-engagement, functionally classifying them as co-stimulatory agonists. By leveraging the unique properties of VHHs and their precise engineering, these co-stimulatory agonists hold significant promise for developing safer and more effective T cell immunotherapies, particularly for combating immunosuppressive environments like those found in solid tumors. Based on high expression yield, diverse binding affinity to CD28 on the cell surface, CDR sequence diversity, and strong agonism, five major VHH candidates were selected from this group for further application evaluation.

To evaluate the application potential of the selected anti-CD28 VHH, we constructed Tri-TCEs by integrating the anti-CD28 VHH module into a Bi-TCE backbone targeting CD3 and DLL3. The resulting Tri-TCEs were expressed in mammalian cells and purified via a streamlined two-step chromatography process. Analytical SEC-HPLC confirmed a monomeric purity exceeding 95%. Notably, the molecule demonstrated excellent biophysical stability: for example, Tri-TCE-VHH38, after incubation at 37 °C for two weeks, the aggregate content remained below 5%, indicating robust thermal and colloidal stability (**Figure 4C**). These favorable physicochemical attributes align with key biopharmaceutical developability criteria and support its potential for further clinical translation.

In multispecific antibodies, steric hindrance between modular components can potentially compromise their respective functions. Our binding analyses revealed that the anti-CD3 domain within the Tri-TCE remained largely unaffected, with minimal shifts in EC_50_ values. In contrast, the binding affinity of the anti-CD28 VHH modules to membrane-anchored CD28 exhibited some variation between the VHH-Fc fusion protein and Tri-TCE molecules, likely attributable to differences in epitope accessibility and spatial constraints. Furthermore, all Tri-TCE constructs demonstrated stronger binding to T cells than the Bi-TCE control, indicating that the anti-CD28 VHH potentiate the avidity of anti-CD3 antibodies to T cells. Furthermore, functional characterization confirmed that both Tri-TCE-VHH38 and Tri-TCE-VHH75 mediated more potent *in vitro* cytotoxicity compared to the Bi-TCE. Among them, Tri-TCE-VHH38 mediates T cell cytotoxicity in a DLL3-dependent manner, with no killing activity observed against DLL3-negative RKO-E6 cells (**Supplementary Figure 3**). This demonstrates that the Tri-TCE format, despite potential steric modulation of individual binding domains, confers a stronger overall capacity to activate T cells, underscoring the therapeutic advantage of integrating a CD28 costimulatory signal.

Next, we further explored the structural basis for the different agonistic activity characteristics exhibited by VHH38 and TGN1412. The mechanistic basis for TGN1412’s superagonistic activity lies in its unique binding properties. It binds specifically to the C’’D loop of CD28, which enables direct, TCR independent T cell activation. This activity is potentiated by its bivalent IgG4 structure and FcγR IIb mediated crosslinking on the cell surface. Our epitope mapping studies, combining antigen alanine scanning and structural modeling, reveal that the VHH38 binds an epitope encompassing the C and C’ *β*-sheets as well as the C’’D loop of CD28, demonstrating a significant overlap with the TGN1412 binding site. Despite this overlapping epitope, and in sharp contrast to TGN1412, the VHH38-Fc fusion protein does not exhibit superagonistic activity, as confirmed in our Jurkat-Luc reporter assays, where it activated T-cells only in the presence of an anti-CD3 signal. A detailed comparison of the binding modes offers a compelling explanation for this functional difference. TGN1412, as a bivalent IgG, engages the CD28 molecule in a ​near-vertical orientation. This topology, particularly when combined with FcγR-mediated crosslinking, is believed to promote intense, non-physiological clustering of CD28 receptors on the T cell surface, thereby triggering uncontrolled signaling. In contrast, our structural model predicts that VHH38 likely binds laterally, monovalently and parallel to the *β*-sheet of CD28. To further support our hypothesis regarding the lateral, monovalent binding geometry, we performed a comparative in silico simulation. We utilized AF3 to model the binding of CD28 with (i) another representative VHH from our screening and (ii) the well-known anti-CD28 scFv (derived from the TGN1412 superagonist). The aligned 3D models clearly illustrate that while the scFv engages the side of the CD28 homodimer, our selected VHHs consistently favor a unique lateral geometry, maybe structurally hindering superagonistic lattice formation (**Supplementary Figure 4**). This unique geometry is unlikely to produce the extensive CD28 aggregation induced by TGN1412. These findings provide a reasonable structural basis for the favorable safety profile of VHH38 when incorporated into the Tri-TCE. Our predictive model is strongly anchored by our empirical Alanine scanning data, which biologically validated the critical interacting residues predicted by AF3 at the lateral interface. We also agree that empirical validation via X-ray crystallography or Cryo-EM will be the ultimate standard to definitively confirm our structural hypothesis in future studies.

The high degree of overlap of VHH binding epitopes originates from the sequence characteristics of CD28 molecules across species. Building upon the identification of VHH38, we employed the same alanine scanning and structural analysis pipeline to characterize additional anti-human CD28 VHHs isolated from the same library. Strikingly, these VHHs were found to share a highly similar binding epitope, primarily localized to the C/C’ *β*-sheets and the C’’D loop of CD28, overlapping with that of VHH38. To investigate the immunological basis for this focused epitope targeting following camel immunization, we performed a comparative sequence analysis of human and camelid CD28 immunoglobulin variable (IgV)-like domains. The two sequences share 84% identity at the amino acid level. However, a key structural divergence was identified: three spatially adjacent, solvent-exposed polar residues in the human CD28 epitope—Tyr51, Tyr61, and Lys63—are substituted by Ser, His, and Ser, respectively, in camel CD28. This localized sequence variation within an otherwise conserved region likely rendered this human-specific epitope highly immunogenic in the camel, providing a structural explanation for the predominant selection of VHHs that target this particular region.

In summary, we have identified VHH38 as a promising ​co-stimulatory module​ for Tri-TCE, based on its distinct ​side-binding mode​ to CD28 and its strict ​TCR dependent agonistic profile, which collectively contribute to a favorable safety and efficacy profile. While our *in vitro* data (**Figure 2D**) confirm that these VHHs strictly require Signal 1 for T cell activation, future studies utilizing structural controls lacking the CD3-engaging arm will be valuable to formally evaluate the absence of cytotoxicity driven solely by CD28-tumor bridging. While our *in vitro* data demonstrate the potent and costimulatory-restricted profile of the identified anti-CD28 VHHs within a Tri-TCE format, we acknowledge the limitations of the current study. The complex dynamics of the tumor microenvironment, including spatial constraints, tumor penetration, and long-term T cell exhaustion, cannot be fully recapitulated *in vitro*. Therefore, comprehensive *in vivo* evaluations using humanized PBMC-engrafted xenograft models are warranted in our future studies to definitively establish the *in vivo* safety, efficacy, and pharmacokinetic profiles of these promising molecules. In parallel, we plan to develop ​CD28/TAA bispecific antibodies​ for use in combination with existing BiTEs, a strategy that will allow direct comparison between a ​combination regimen​ and an ​integrated TriTE molecule​ in terms of efficacy, safety, and pharmacokinetics. Furthermore, we intend to extend this modular platform to target other solid-tumor antigens, such as members of the ​tight junction protein family (Claudin) and members of the epidermal growth factor receptor family (HER)​. This expansion will help to systematically assess the broad applicability and therapeutic potential of CD28-costimulated TriTEs across diverse solid tumor types.

## 5 Conclusion

This study successfully identified and characterized a diverse panel of co-stimulatory agonistic, anti-human CD28 VHHs that function as versatile and effective costimulatory modules for T cell immunotherapies. Through functional screening and structural characterization, we identified VHH38 as a lead candidate, which was incorporated into a Tri-TCE targeting CD3, CD28, and DLL3. The resulting molecule, Tri-TCE-VHH38, demonstrated favorable developability properties and exhibited superior efficacy in T cell binding and tumor cell killing compared to a Bi-TCE control.

Epitope mapping and structural analysis revealed that VHH38 engages a classic CD28 agonist epitope through a side-binding paratope. This unique binding geometry provides a mechanistic basis for its ​TCR dependent conventional costimulatory agonistic profile, distinguishing it from superagonistic antibodies. This work establishes a foundation for rationally incorporating CD28 co-stimulation into next generation multispecific therapeutics, offering a promising strategy to improve T cell-based treatments for cancer and other immune mediated diseases.

## Data Availability

The raw data supporting the conclusions of this article will be made available by the authors, without undue reservation.
