# Supplementary figures and images for: Discovery of co-stimulatory anti-CD28 VHHs for developing cancer immune therapeutic anti-tumor/CD3/CD28 trispecific T cell engager
Source: Front Immunol. 2026 Apr 17;17:1812063. doi: 10.3389/fimmu.2026.1812063 (PMC13133060; doi:10.3389/fimmu.2026.1812063)

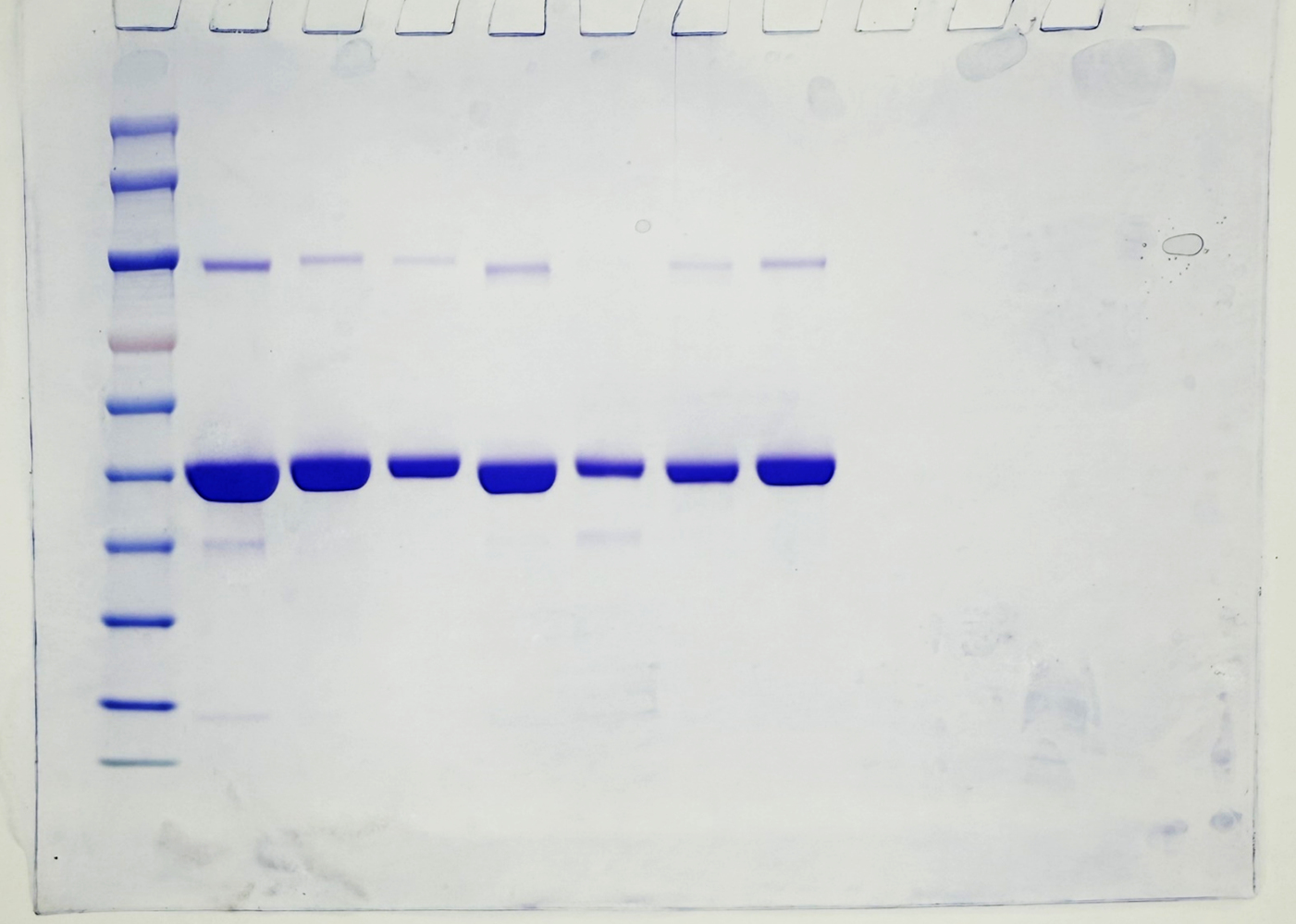

Supplement: Supplementary file 1 [file DataSheet1.zip › Frontiers manuscript-1812063-Fig/Fig 2A-1.png]

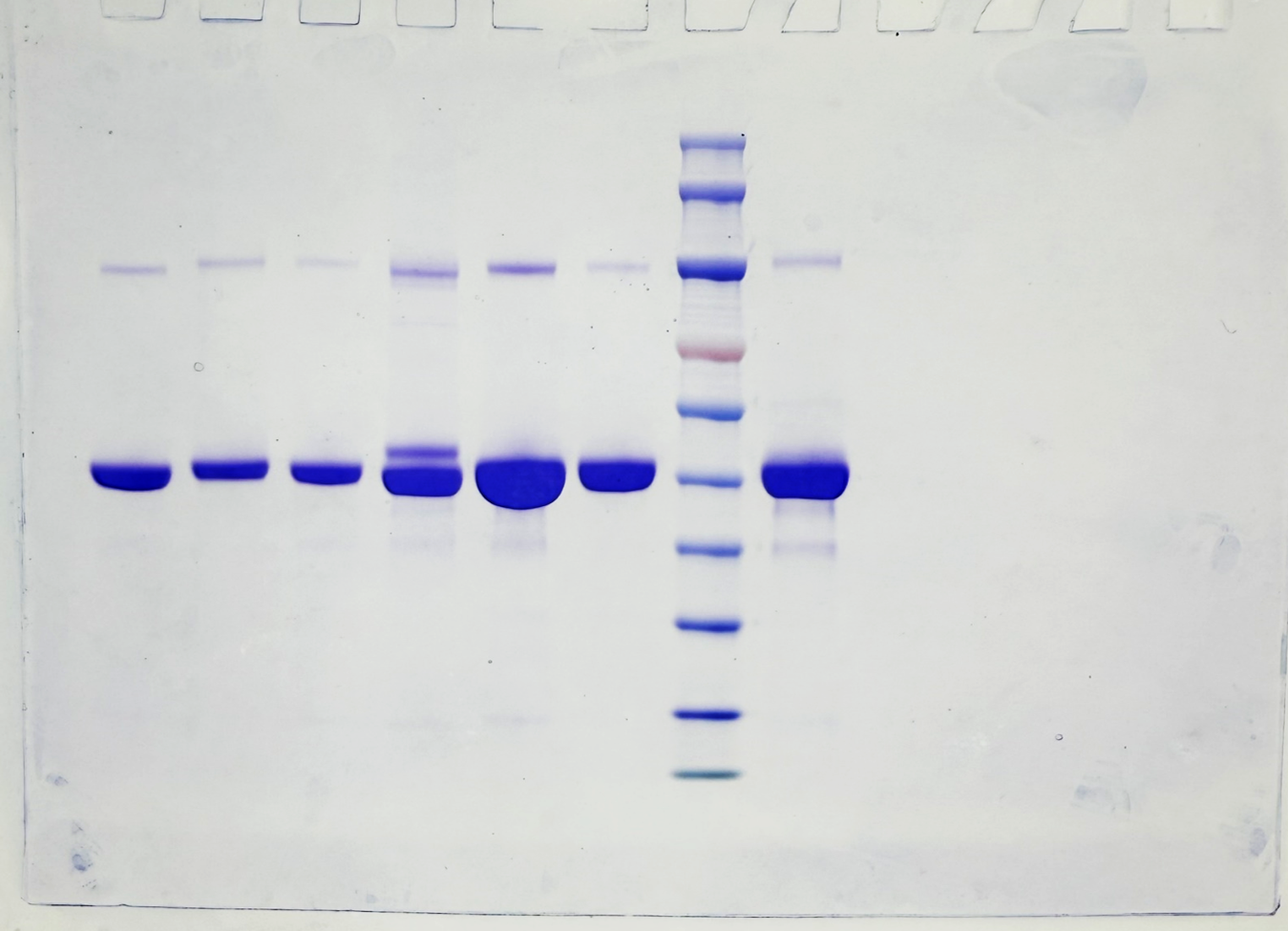

Supplement: Supplementary file 1 [file DataSheet1.zip › Frontiers manuscript-1812063-Fig/Fig2A-2.png]

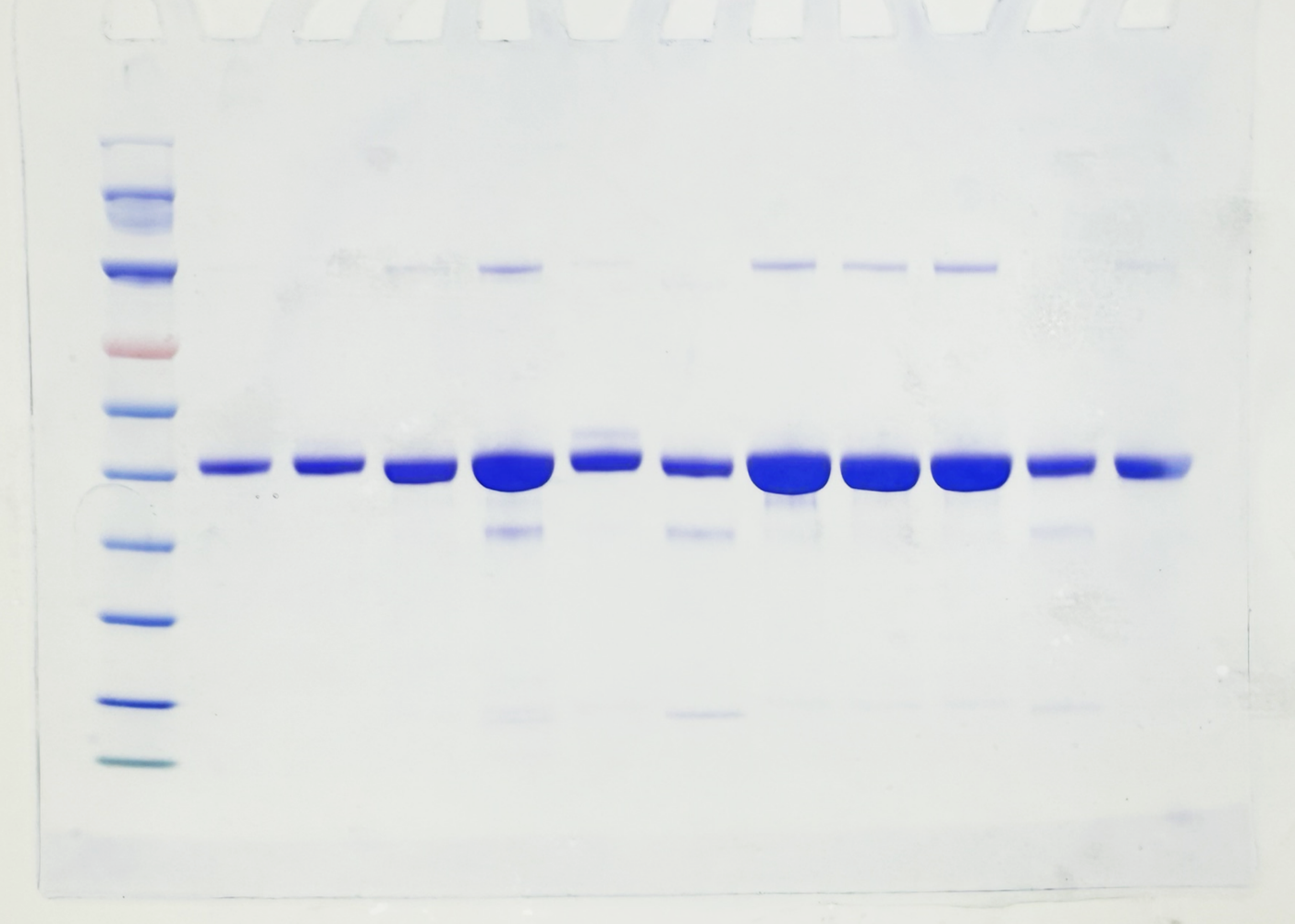

Supplement: Supplementary file 1 [file DataSheet1.zip › Frontiers manuscript-1812063-Fig/Fig2A-3.png]

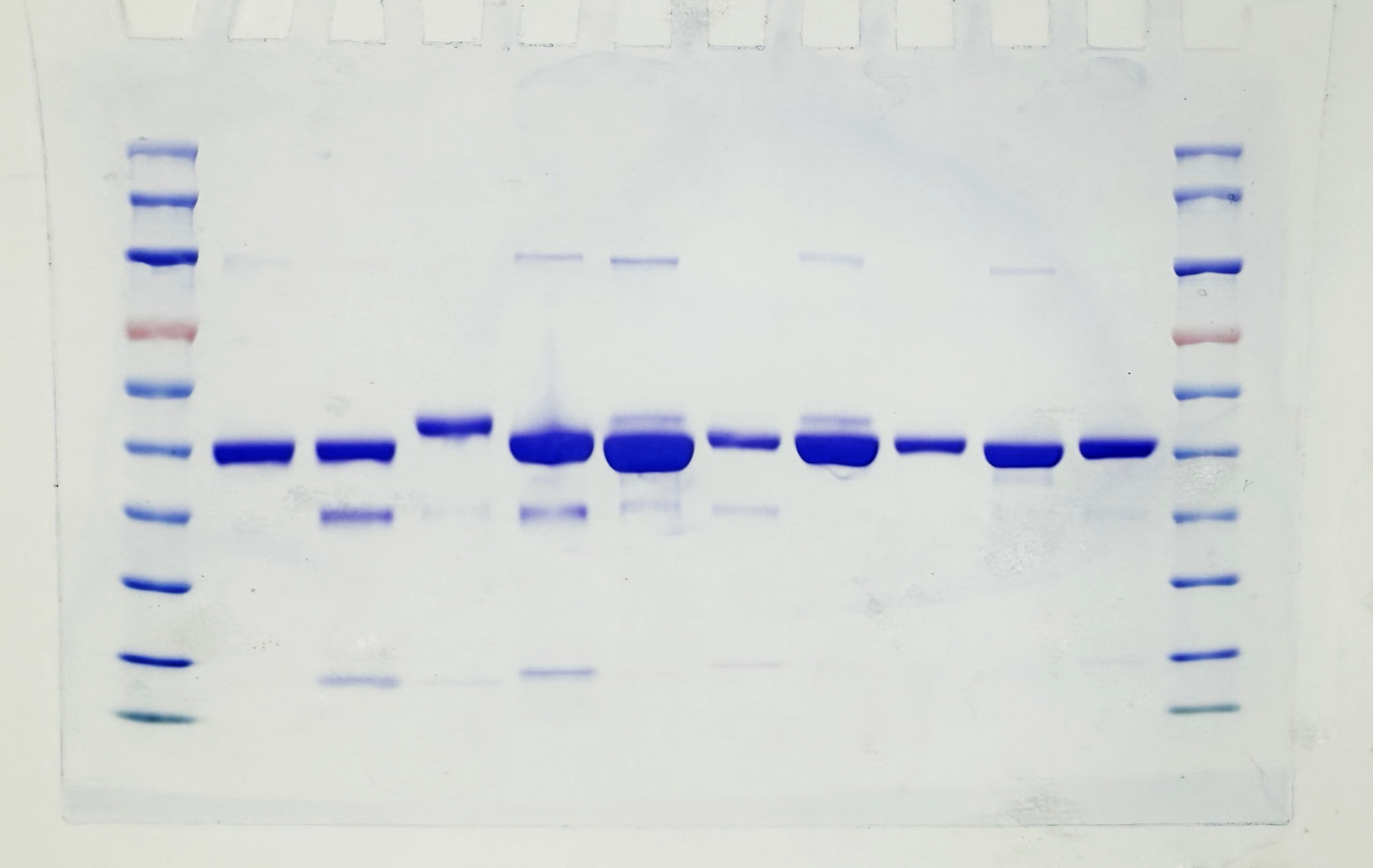

Supplement: Supplementary file 1 [file DataSheet1.zip › Frontiers manuscript-1812063-Fig/Fig2A-4.png]

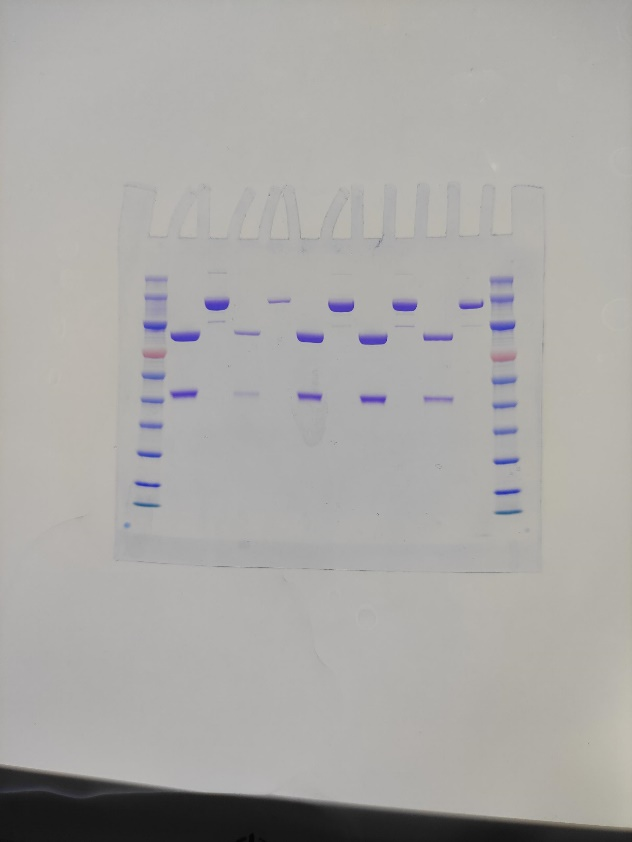

Supplement: Supplementary file 1 [file DataSheet1.zip › Frontiers manuscript-1812063-Fig/Fig3B-1.png]

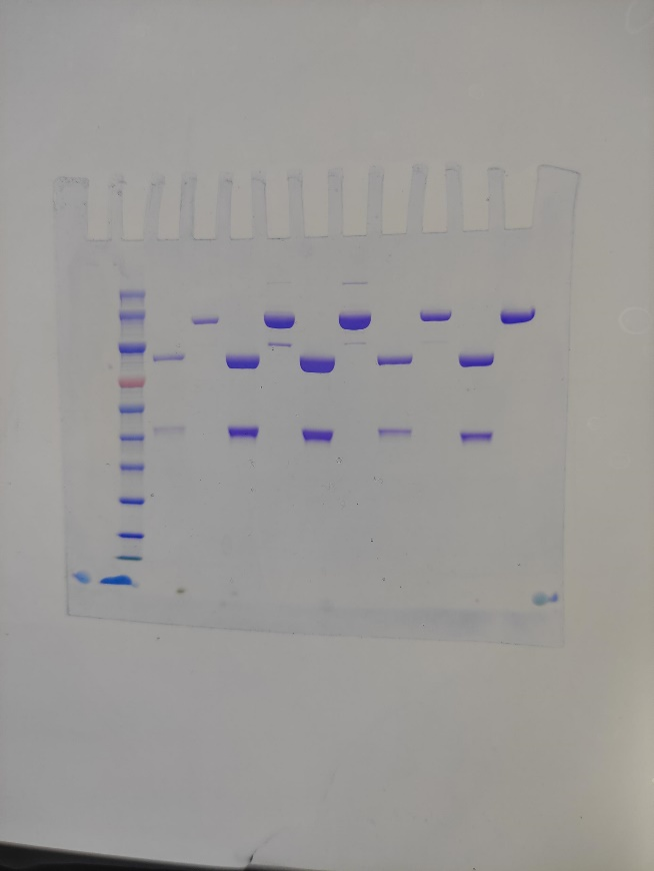

Supplement: Supplementary file 1 [file DataSheet1.zip › Frontiers manuscript-1812063-Fig/Fig3B-2.png]
