# Supplementary material for: Discovery of co-stimulatory anti-CD28 VHHs for developing cancer immune therapeutic anti-tumor/CD3/CD28 trispecific T cell engager
Source: Front Immunol. 2026 Apr 17;17:1812063. doi: 10.3389/fimmu.2026.1812063 (PMC13133060; doi:10.3389/fimmu.2026.1812063)

**Supplementary Material**

**Discovery of co-stimulatory anti-CD28 VHHs for developing cancer immune therapeutic anti-tumor/CD3/CD28 Trispecific T cell Engager**

Hui Wang^a^, Rui Zhao^b^, Mengmeng Liu^b^, Tianzhi Jiang^a^, Di Sun^b^, Lida Chi^b^, Yonglin Huang^b^, Zhenghui Lu^a^*, Yueli Yun^a^*, Xiangbin Wang^b^*

^a^State Key Laboratory of Biocatalysis and Enzyme Engineering, School of Life Sciences, Hubei University, Wuhan 430062, People's Republic of China

^b^Beijing Scipromed biotech Co., Ltd., Beijing 102299, People's Republic of China


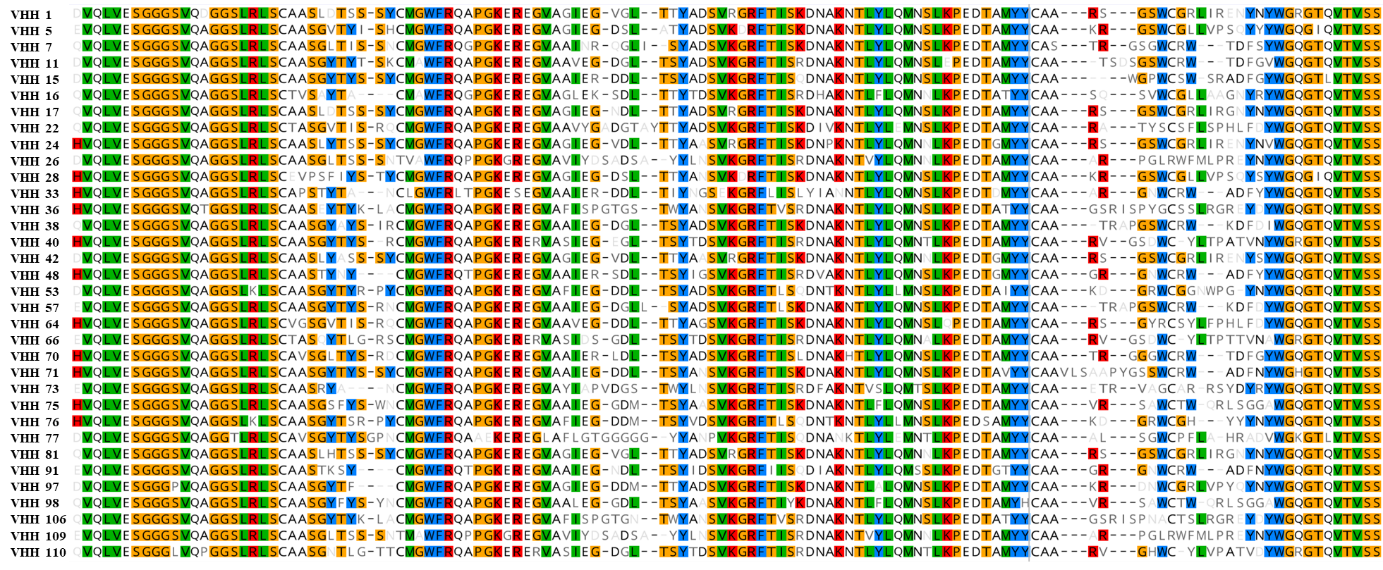


**Figure S1. The sequence of αCD28 VHH.**


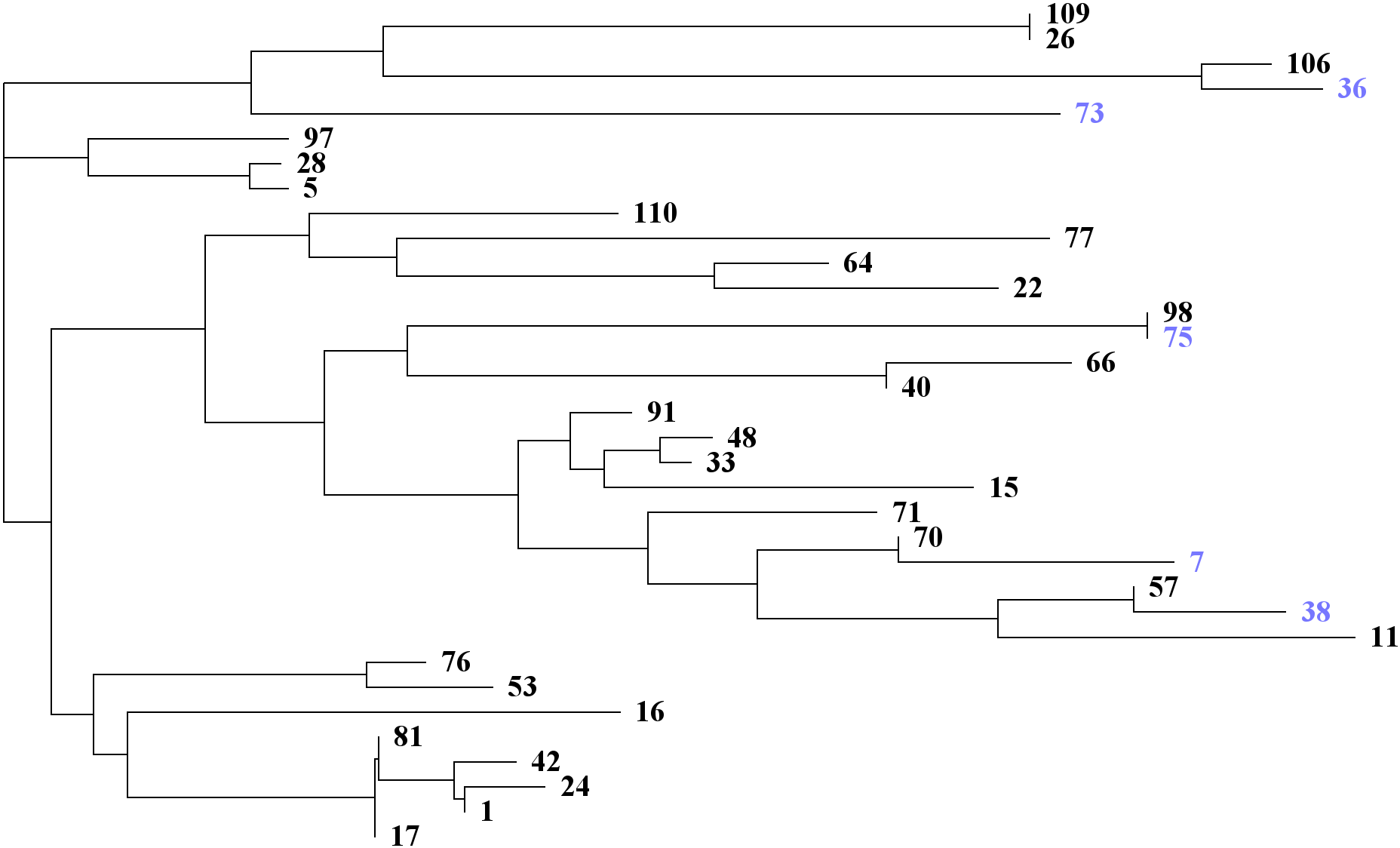


**Figure S2. Cluster analysis of VHH CDR3 sequences.** Clustering performed using MAGA.

**Figure S3. Tumor antigen-dependent cytotoxicity.** Cytotoxic activity of PBMCs against SHP77 cells or RKO-E6 (a colon cancer transgenic cell line that does not express DLL3) in the presence of the indicated antibodies. The tumor target cells cocultured with human PBMCs at an effector-to target ratio of 5:1. Effector cytolytic activity was assessed after 48 hours. Data shown here are mean ±SD values from three independent triplicates values. The results are representative of at least three independent experiments.


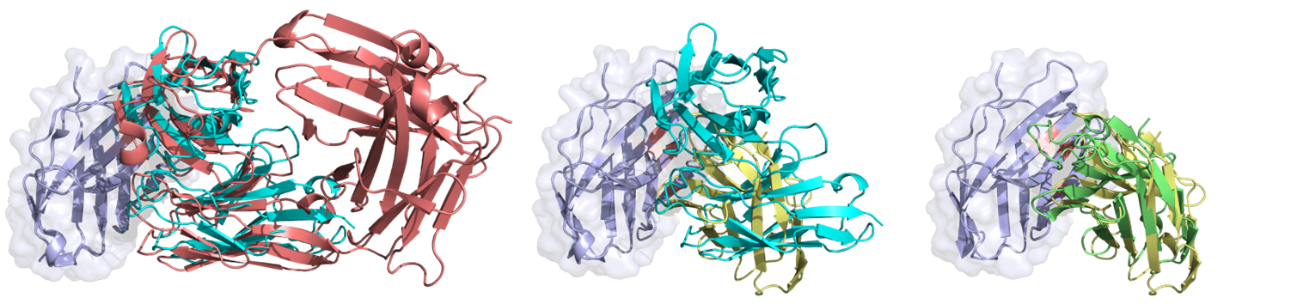


**Figure S4. Structural superposition.** Left panel:​ Superposition of the crystal structure of CD28/TGN1412-Fab (PDB: 1yjd) and the AF3-predicted structure of the CD28/TGN1412-scFv complex. The CD28 is shown as a light blue cartoon with its surface displayed; TGN1412-Fab (PDB: 1yjd) is in red cartoon, and TGN1412-scFv is in cyan cartoon. Middle and right panels:​ CD28 is labeled in the same manner as in the left panel, with TGN1412-scFv in cyan cartoon. The VHH38 is shown in yellow cartoon, and VHH36 in green cartoon.

**Table S1. Expression yield of nanobodies.**


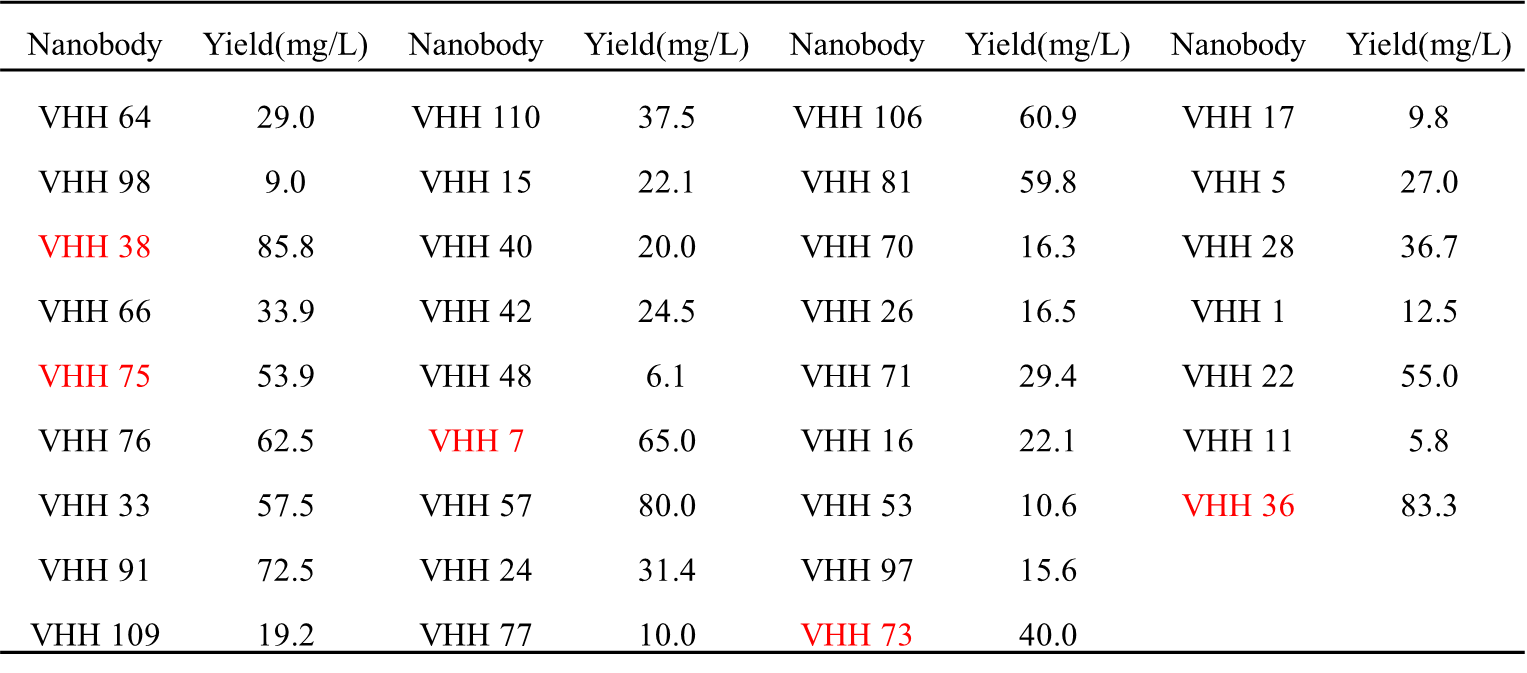

Supplement: Supplementary file 2 [file Table1.docx]
